# Mutual human-robot understanding for a robot-enhanced society: the crucial development of shared embodied cognition

**DOI:** 10.3389/frai.2025.1608014

**Published:** 2025-07-03

**Authors:** Giulio Sandini, Alessandra Sciutti, Pietro Morasso

**Affiliations:** COgNiTive Architecture for Collaborative Technologies Research Unit, Robotics, Brain and Cognitive Sciences Research Unit – Italian Institute of Technology, Genoa, Italy

**Keywords:** embodied artificial intelligence, embodied cognitive science, enaction theory, simulation theory of cognition, developmental psychology, ecological psychology, prospection, extended mind hypothesis

## Abstract

The conception of autonomous, intelligent, collaborative robots has been the subject of science fiction rather than science in the second half of the previous century, with practical applications limited to industrial machines without any level of autonomous, intelligent, and collaborative capacity. The new century is facing the challenge of pressing industrial and social revolutions (4, 5, 6, …) with the prospect of infiltrating robots in every sector of human society; however, this dissemination will be possible if and only if acceptable degrees of autonomy, intelligence, and collaborative capacity can be achieved. Scientific and technological innovations are needed within a highly multidisciplinary framework, with a critical integration strategy and functional characterization that must ask a fundamental question: the design of autonomous, intelligent, collaborative robots should aim at a unified single template to be mass-produced including a standard setup procedure for the functional adaptation of any single prototype, or should the design aim at “baby” robots with a minimal set of sensory-motor-cognitive capabilities as the starting point of a training and educational process in close connection with human companions (masters, partners, final users)? The former alternative is supported by EAI, i.e., the Embodied variant of the Artificial Intelligence family of computational tools based on large foundation models. The latter alternative is bio-inspired; namely, it attempts to replicate the computational structure of Embodied Cognitive Science. Both formulations imply embodiment as a core issue. Still, we think this concept has a markedly different meaning and practical implications in the two cases, although we are still far away from the practical implementations of either roadmap. In this opinion paper, we explain why we think the bio-inspired approach is better than the EAI approach in providing a feasible roadmap for developing autonomous, intelligent, collaborative robots. In particular, we focus on the importance of collaborative human-robot interactions conceived in a general sense, ranging from haptic interactions in joint physical efforts (e.g., loading/unloading) to cognitive interactions for joint strategic planning of complex tasks. We envision this type of collaboration only made possible by a deep human-robot mutual understanding based on a structural equivalence of their embodied cognitive architecture, based on an active, first-person acquisition of experience rather than a passive download of third-person knowledge.

## Introduction

The conception of autonomous, intelligent, collaborative robots has been the subject of science fiction rather than science in the second half of the previous century, with practical applications limited to industrial machines without any level of autonomous, intelligent, and collaborative capacity. The new century faces the challenge of pressing industrial and social revolutions (4, 5, 6, …) with the prospect of infiltrating robots in every sector of human society. Still, this dissemination will be possible if and only if acceptable degrees of autonomy, intelligence, and collaborative capacity can be achieved. Scientific and technological innovations are needed, pursued within a highly multidisciplinary framework, with a critical convergent strategy and functional characterization that must ask a fundamental question: the design of autonomous, intelligent, collaborative robots should aim at a unified single template to be mass-produced, including a standard setup/tuning procedure for the functional adaptation of any single prototype, or should the design aim at baby robots with a minimal set of sensory-motor-cognitive capabilities as the starting point of a training and educational process in close connection with human companions (masters, partners, final users)? Considering that both alternatives are decades from an actual implementation/application level, we suggest weighing the pros and cons of the different options and the robustness of their founding bases beyond the strong and/or excessive acclamation of AI technologies.

## Knowledge, cognition, intelligence, wisdom

The 2024 Nobel Prizes in Physics and Chemistry awarded to two artificial intelligence scientists have highlighted the social and economic expectations for a scientific methodology based on big data and big computational power, whose scientific substance, in the framework of the modern scientific method established by Galilei and Newton, is still to be understood and still to be proven. There is no doubt that commercially available large language models (LLMs) or vision language models (VLMs) would pass the Turing test, exhibiting the machine’s ability to participate in plausible, natural language conversation. In particular, recent experimental results show that GPT-4.5 can pass formalized versions of such test when suitably prompted, with human judges identifying them as human in over 70% of cases—even more frequently than actual human participants (Jones and Bergen, 2025).

We should remember that the test was initially called the imitation game, and as such it could be considered more as a humanlikeness test rather than a direct test of intelligence. Moreover, the only aspect tested is verbal ability, which, by itself, is not the only source for acquiring and organizing knowledge and is certainly not related to the acquisition and organization of goal-driven motor abilities required in robotics. At the same time, there is no doubt that AI models can accelerate the search of complex problem spaces and thus can be used as powerful tools for discovering regularities and hidden properties in large data sets, e.g., for predicting proteins complex structures.

Moreover, the efficiency of the new computational tools does not imply that they characterize a new and superior scientific paradigm, as proposed by AI enthusiasts (Martin and Mani, 2024; Xu et al., 2024) who expect foundation models in generative AI to evolve into a new scientific domain: the crucial innovation would be to overcome the cognitive limitations of human minds, thus achieving reasoning and mental abilities far exceeding those of well-educated humans, including the most distinguished scientists as well as Nobel prize winners. In other words, the evolution of large AI models is expected by AI enthusiasts to quickly achieve a form of general AI (AGI) at the highest level of human intelligence, and then overcome it, ultimately becoming a superhuman omniscient sage, capable of “wisdom,” on top of immense amounts of encyclopedic knowledge; without attempting to define in a cogent way such challenging concept, either from the natural or artificial point of view, the jump from intelligence to wisdom is difficult to explain and justify.

In our opinion, the expectation of a future form of “artificial wisdom” on the side and the top of a supreme form of artificial intelligence is somehow illogical and frightening at the same time: in particular, we should consider the well-known ethical problems of AI expressed by many, including one of the two Nobel prize winners (Geoffrey Hinton), interviewed by Heaven (2023). Although the ethical issue is outside the focus of this paper on cognitive robotics, the scientific and technological rationale of an envisaged artificial wisdom needs to be addressed at the beginning of this article, which is based on the crucial role of embodied cognition for human-robot interaction and collaboration principles.

Knowledge, intelligence, and wisdom are related but distinct concepts about human nature, which a standard template cannot capture because each individual is somehow unique, i.e., an exception concerning any conceivable standard, and thus, human nature, in general, is paradoxical, contradictory, and subject to continual change. An inteligent system in the framework of current AI is mainly a system with problem-solving ability that implies the knowledge of large sets of facts and rules. Human wisdom is characterized by several potentially conflicting components (Jeste and Lee, 2019), such as social decision-making, a value system, emotional regulation, prosocial behaviors, self-reflection, acceptance of uncertainty, decisiveness, fusing knowledge with experience, insight, good judgment, etc. Moreover, wisdom has a fundamentally social function, namely, to suggest and induce people to consider the consequences of their actions to themselves and society in the framework of a value system, and there is experimental evidence that the relationships between intelligence and wisdom in individuals is far from linear (Glück and Scherpf, 2022). Thus, moving from the natural to the artificial domain, there is no solid ground to believe that betting on intelligence to achieve wisdom is a promising evolutionary outline. Modern history, marked by the Renaissance and illuminism, which nurtured and consolidated the emergence of the scientific paradigm as an undisputed methodological standard, is a clear witness of the erratic evolution of the shared understanding of the concept of wisdom: in any case, wisdom appears as a changing work in progress, as something to be decided and conquered by a community of individuals, within some democratic framework, not the exceptional individual capability to be evaluated in a competition, as a chess game. Consequently, the expectation of autonomous artificial wisdom as the emerging asymptotic property of higher and higher versions of AI foundation models appears illogical and misleading, scientifically and socially.

In short, we believe that considering advanced forms of AI as a new scientific paradigm is wrong. However, such technologies will be a powerful tool in developing the fourth industrial revolution and beyond. Moreover, there is a predictable “saturation” of the AI-driven futuristic scenarios determined by the maturation of new computing scenarios such as quantum computing (Morasso, 2023; Gill et al., 2024) and/or wetware/organic computing (Jordan et al., 2024), thus overcoming the limitations of the digital framework that characterizes the current computational formulation of AI. Quantum computing is expected to address the quantum effects that underlie dynamic interaction at the nano-scale in biology, opening the door to an entirely new understanding of the self-organizing processes that characterize the organization and the development of the central nervous system, thus supporting the evolution of natural intelligence. Moreover, a crucial difference between the all-digital scenario and the scenario based on quantum/wetware computing is the energetic consumption, i.e., the energetic frugality intrinsic in the non-digital or only partially digital future scenarios.

On the other hand, the surprising performance of recent foundation models, such as large language models (LLMs) or vision language models (VLMs), hides the fact that these models are essentially passive: they are trained based on vast amounts of data, and thus are intrinsically unable to provide the robot’s body with specific inference capabilities, necessary for identifying in any given task the crucial and typically small set of information and combination of actions that make the difference between success and failure or wise versus foolish behavior.

## AI vs. embodied cognition

AI is a disembodied computational process conceived to conduct all sorts of imitation games, starting with the Turing test (Turing, 1950). The issue of embodiment is called on stage if an AI agent is requested to act, i.e., the agent is supposed to answer questions coming from the physical world, and the answers should somehow produce effects in the same world. Thus, the cognitive agent must have a body, which includes sensors for detecting and evaluating what happens in the environment and actuators for generating physical effects within a well-defined time framework: in real-time, delayed-time, or intermittent-time. Probably, the first example of a minimal embodied AI agent was proposed by Braitenberg (1984) describing *Vehicles*, namely a class of simple, autonomous moving agents where simple, conceptually analog, wired schemes implemented the process between visual sensors and motor actuators. Depending on the chosen scheme and the structure of the environment, the vehicle could show a variety of complex behaviors that may appear flexible, adaptive, goal-directed, and even intelligent, although in a nutshell scale, without any specific cognitive processes.

This minimalistic approach to what is now known as EAI (Embodied AI) was expanded by Brooks (1991), who proposed that there is no need for complex algorithms or internal representations for producing intelligent behaviors of autonomous agents because the key source of adaptive dynamics is the direct physical interactions of the agent with its environment. Since such interaction is made possible by the agent’s body, Brooks concluded that Intelligence must have a body and suggested calling it “embodied intelligence.” This *embodiment hypothesis* was further elaborated upon by Smith and Thelen (1994), Pfeifer and Scheier (2001), and Smith (2005), among others, ending up with the current understanding that EAI is a variety of AI that integrates artificial intelligence into physical entities like robots, endowing them with the ability to perceive, learn from, and dynamically interact with their environment: in other words, the explicitly stated goal of EAI is to build General-Purpose Robots via Foundation Models (Hu et al., 2023), namely “robots that operate seamlessly in any environment, with any object, and utilizing various skills to complete diverse tasks.” The problem is how to integrate the rationale of data-driven foundation models in the sensory-motor-cognitive structure of a robot, covering the large variety of functions related to perception, prospection, task planning, and action generation: the combined “space” of environments, tasks, and actions is virtually infinite, and attempting to sample it to train a set of foundation models that may encapsulate the cognitive capabilities expected of a generally intelligent robot looks like a hopeless goal, at least in an open environment and in the framework of bounded resources.

On the other hand, we should consider that the same goal of conceiving and designing robots with general intelligence has been investigated well before EAI under the label of Cognitive Architectures for Cognitive Agents. This research field has been very active for several decades, as reported by a recent review (Kotseruba and Tsotsos, 2020), considering tens of projects at different levels of development. However, we are still far away from some standard framework. Among the well-developed prototypes, some architectures focus on modeling human cognition in general as a unified theory of cognition, like SOAR (Laird et al., 1987), ACT-R (Anderson, 1996), CLARION (Sun, 2007), and others aimed explicitly at developmental robotics as iCub (Vernon et al., 2007, 2011) or the cognitive software framework of humanoid robotscognitive robotics, such as ISAC (Kawamura et al., 2008), ArmaX (Vahrenkamp et al., 2015), and CRAM (Beetz et al., 2023). In different manners, such prototypes integrate essential cognitive functions for autonomous cognitive agents (e.g., active perception, purposive action, perceptual inference, learning, adaptation, anticipation, prospection, motivation, attention, action selection, memory, reasoning) with a hybrid combination of computational tools, including symbolic tools (based on logic-based programming and the use of rules and axioms to make inferences and deductions) and sub-symbolic ones, similar to the neural networks of the foundation models of EAI.

However, in both approaches considered above, the embodiment issue plays only a minor role, namely the integration of input–output peripherals with a reasoning/inference machinery of different complexity levels: in the case of the Vehicles model (Braitenberg, 1984), the computational machinery is a simple hand-wired electronic circuit; in the case of CRAM (Beetz et al., 2023) the computational machinery includes self-programmability entailed by physical symbol systems, a plan language for generalized action plans, implicit-to-explicit manipulation, generative models, digital twin knowledge representation, and narrative-enabled episodic memories; in the case of the envisaged innovation toward general-purpose robots via foundation models (Hu et al., 2023) it is expected to apply both existing vision and/or language foundation models already modified for robotic applications (Ahn et al., 2022; Chen et al., 2023) as well as models specifically developed for robotic functions (Brohan et al., 2023a, 2023b) counting on their potential generalization ability across different tasks and even embodiment schemes. In this view, embodiment is somewhat limited and reduced to a one-way flow of information from the sensory periphery toward more remote areas of the brain and then back to the motor periphery. More generally, there is ground to doubt to which extent EAI is really embodied (Hoffmann and Patni, 2025).

In contrast to the minimal utilization of the embodiment concept that characterizes EAI, we should consider an alternative view, namely a form of Embodied Natural Intelligence based on cognitive neuroscience, in particular the subfield known as embodied cognition (Varela et al., 1991; Clark, 1997, 1998) which emerged in the nineties in opposition to the Cartesian dualism and, more recently, to cognitivism and computationalism. Embodied models of cognition are opposed to the disembodied Cartesian model, according to which all mental phenomena are non-physical and thus not influenced by the body, as well as to EAI models where embodiment is limited to one-way interaction between brain, body, and environment. By embodiment, the supporters of embodied cognition refer to the circular, bi-directional interaction where the body allows the brain to physically interact with the environment to accumulate and distill personal experiences, driving the formation and evolution of the agent’s cognition. An account of this process is proposed by Varela et al. (1991) as enaction theory, whereby cognitive processes incorporate sensations into a sensorimotor loop, through which active experience of the environment is realized (“enacted”). In this framework, the goal is not to learn the model of the world through interaction but to learn the model of the interaction between the agent and the world. Such a view about embodied cognition, based on the working hypothesis that cognitive processes are deeply rooted in the body’s bi-directional interactions with the world, is summarized by Wilson (2002) into six claims about the fundamental features of human embodied cognition which have a direct computational relevance: (1) situated-ness, (2) time-pressured-ness, (3) exploitation of the intrinsic dynamics of the environment, (4) integration of the environment in the cognitive architecture, (5) cognition is mainly an online, action-oriented process; (6) even offline cognition is body based. The experimental baseline to support such claims is diverse and includes the following lines of evidence: ideo-motor theories of perception (Prinz, 1987); the developmental psychology of Piaget (1952) who traced the evolution of cognitive levels from the consolidation of sensorimotor abilities up to higher levels; the ecological psychology of Gibson (1977) who characterized active perception as the discovery of potential interactions with the environment, i.e., affordances; the linguistic decomposition of abstract concepts in terms of qualitative explanations based on bodily metaphors (Lakoff and Johnson, 1999); the sociocultural theory of Vygotsky (1978) emphasizing the role of social interaction in shaping cognitive development.

Despite all this evidence in favor of the view that the mind must be understood only in the context of its relationship with a physical body that interacts with the world in an online manner (the embodiment hypothesis), it has been objected that cognitive activities of various nature can take place as well when the brain is decoupled from any immediate interaction with the environment, i.e., in an offline manner. This coexistence of online and offline operational modes of human cognitive activities contradicts the embodied cognition hypothesis, which claims to characterize the whole of cognition, not only a part of it. However, this is only an apparent paradox if we associate the online vs. offline antinomy (related to the interaction of a cognitive agent with the environment) with two additional antinomies, namely actual vs. imagined activities and overt vs. covert actions: these antinomies reflect the computational and neural equivalence between the sensory-motor-cognitive processes involved in the execution of purposive actions and the processes activated for reasoning about virtual actions, for example in the context of the fundamental cognitive function known as prospection. Prospection is the mental simulation of actions to evaluate their potential sensorimotor, environmental, and social effects in the future, thus supporting an informed (and potentially wise) decision-making process (Gilbert and Wilson, 2007; Seligman et al., 2013; Vernon et al., 2015). The experimental evidence about the equivalence stated above comes from the study of motor imagery (Decety, 1996; O’Shea and Moran, 2017) and different forms of a simulation theory of cognition (Decety and Ingvar, 1990; Jeannerod, 2001; Hesslow, 2002, 2012; Grush, 2004; Ptak et al., 2017); an essential part of this theory is that the simulation is performed by the same neural mechanisms as those typically involved in movement execution and perception, although some researchers suggest that simulation (or emulation) of actions is performed by a neural mechanism that is different and separate from brain areas directly involved in movement and perception (Wolpert et al., 1998). In any case, the equivalence between overt and covert actions does not refer only to the geometry of the involved brain areas but also to the timing of the simulated actions in comparison with the executed ones (Shepard and Metzler, 1971; Decety et al., 1989; Decety and Jeannerod, 1995; Karklinsky and Flash, 2015; Gauthier and van Wassenhove, 2016).

The issue about the apparent online vs. offline paradox, related to the timing of purposive action in support of embodied cognition, is further completed if we consider another facet of human cognition, related to the spatial aspect of purposive actions, i.e., the role of cognitive cortical maps: such brain structures, located in the medial temporal lobe, were proposed by Tolman (1948) for understanding flexible behavior in rodents, e.g., foraging patterns by rats in mazes. In humans, it has become evident that, in addition to their function in spatial navigation, cognitive maps are also the backbone of a systematic organization of knowledge in abstract spaces in such a way as to support the learning of higher-level knowledge (Behrens et al., 2018; Bellmund et al., 2018; Bokeria et al., 2021; Qiu et al., 2024): this means that neurons previously identified in cognitive maps for guiding navigation in the physical environment, such as “place cells,” “grid cells” and “head-direction cells” are also likely to support the ability to mentally “navigate” through conceptual spaces for more abstract reasoning tasks.

The double role of cognitive maps, namely the integration in the same brain structure of the “geometrical” aspects of actions at different abstract levels, together with the double role of the brain areas involved in the simulation theory of cognition, for the representation of the “timing,” “kinematic,” and “haptic” aspects of overt as well as covert actions, explains in which sense and how much embodied human cognition is fundamentally embodied in contrast to the minimal degree of embodiment which characterizes artificial intelligence, in general, and EAI in particular.

From the philosophical standpoint, the strong formulation of the nature and organization of embodied cognition is consistent with the *extended mind hypothesis* (Clark and Chalmers, 1998), namely the belief in the fundamental active role of the environment in driving cognitive processes. Learning, one of the mind’s primary functions, emerges from the closed-loop dynamics that link active perception, purposive action, cognition, and dynamically changing environment. In other words, learning and other fundamental cognitive functions as prospection should be understood in the framework of an extended theory of the mind, which includes the changing environment as a part of the mind dynamical model. In the extended mind hypothesis framework, we should also consider that such extension is naturally articulated in two directions: extension to the physical environment and the social environment. In other words, we should assume that the process of mind extension is not innate or genetically coded, although it is based on genetically based mechanisms, but is mainly the product of different developmental processes, articulated in two main streams: (a) the multi-stage theory of cognitive development, starting with the sensorimotor stage (Piaget, 1952); (b) the sociocultural theory of cognitive development (Vygotsky, 1978).

**Figure 1:**
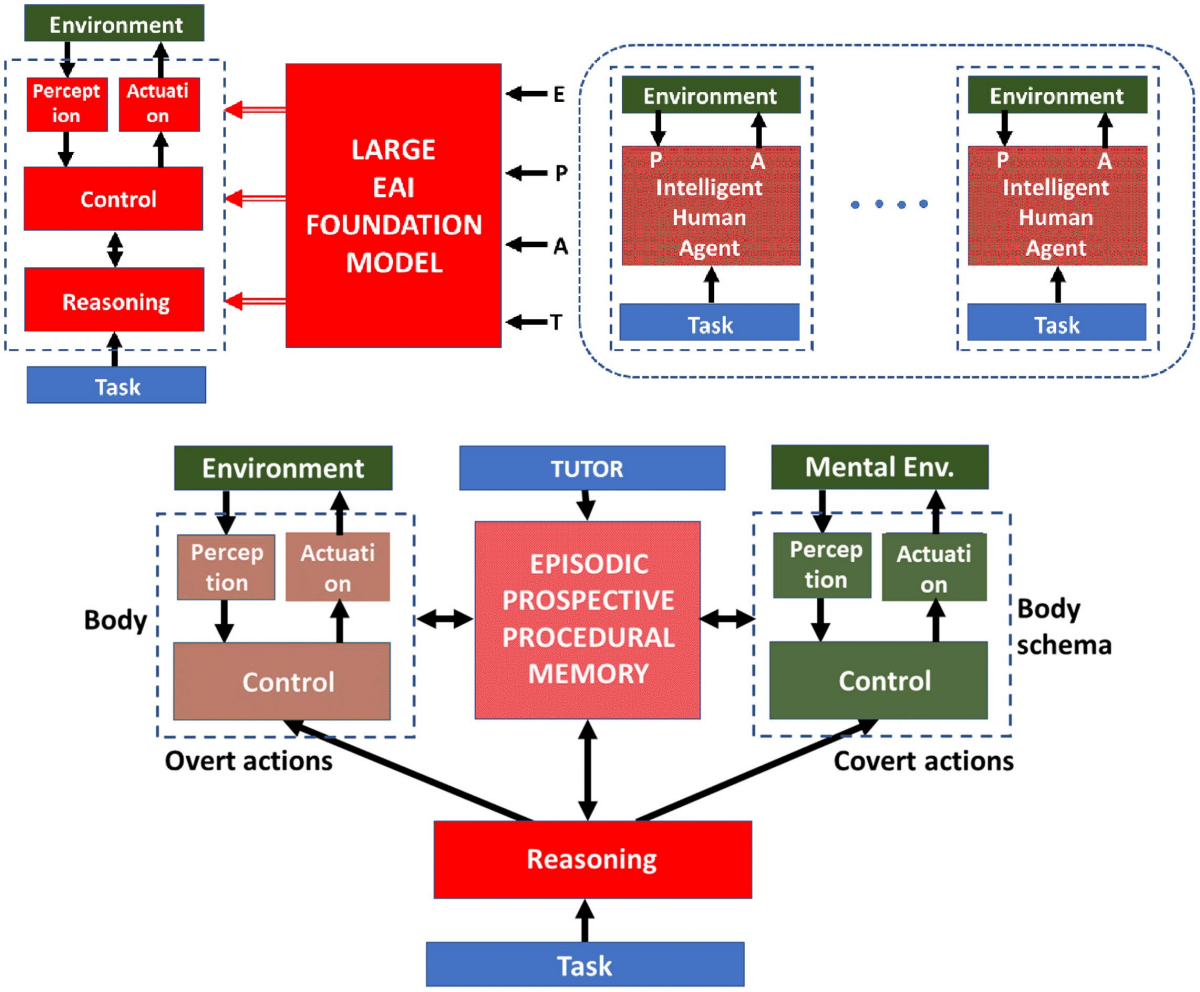
Design principles of autonomous, intelligent, collaborative robots. Summary of the difference between the roadmap based on EAI (top section) and the roadmap based on embodied, developmental cognition (bottom section).

Figure 1 illustrates in a simplified manner the difference between the two roadmaps examined above for the design of autonomous, intelligent, collaborative robots, namely the roadmap based on AI foundation models and the roadmap based on full embodied cognition. The former alternative, in the top panel, shows that the sensory, motor, control, and reasoning processes that are required for carrying out a given task as a function of a given environment are inferred from a large foundation model, trained by the sampled performance of a population of skilled human agents operating in similar situations, i.e., a large dataset of third-person knowledge: the crucial point is that such data are collected with unnecessary high-resolution but with insufficient filtering of “keyframes.” The bottom panel illustrates the main features of the proposed bio-inspired roadmap. In particular, it singles out the crucial features of embodied cognition, based on the accumulation of first-person experience, filtered and organized into a personal episodic/procedural memory, evaluated through a prospective process that combines overt and covert actions using a body model and cooperative interactions with a skilled tutor. Although the body, which is in charge of producing overt actions, and the body-schema, which is supposed to deal with covert (mental) actions, are represented graphically as different blocks, we must remember that, in agreement with the theory on the neural simulation of actions, they incorporate and integrate both functions in the same computational module.

## Embodied cognition and computational frugality

Summarizing the analysis of the previous section, we highlight that human cognition substantiates first-person (autobiographical) human experience, characterized as embedded, enactive, and extended, based on the continually changing, embodied, and affective interactions with the world. In contrast, EAI aims at the design of general-purpose robots via foundation models (Hu et al., 2023) based on large amounts of pre-coded, general-purpose, encyclopedic knowledge, providing third-person (impersonal) experience with a low degree of embodied cognitive interaction. In any case, the goal of EAI is still far away, and possibly the expectation of functionally open-ended, general-purpose, super-intelligent robots is an unreasonable dream. Even EAI enthusiasts (Liu and Wu, 2024) recognize that there is still a lot to do because large foundation models (LLMs and VLMs) may support only a part of the essential cognitive capabilities of autonomous intelligent robots, providing efficient inference capabilities. In the framework based on foundation models, what is lacking is related to the crucial first-person cognitive experience that may allow a robot to be truly autonomous, intelligent, and efficient. In particular, it is suggested that EAI robotics must develop several brand-new cognitive models as the following: an evolutionary learning process driven by the agent’s physical interactions with open environments; multiverse representations of a virtual environment that can effectively emulate the real world, and interact with the EAI systems (Hall et al., 2022); understanding the physical world, such as the concept of gravity, by using intuitive physics models (Piloto et al., 2022).

In general, at the current level of development and understanding, EAI-based robots are likely to face a kind of deep “personality conflict,” namely the conflict between the third-person, disembodied, offline-trained foundation models that should provide the central core of the cognitive capabilities and the first-person, online interaction with the environment and the associated training processes. There is no guarantee that the two coexisting paradigms can avoid conflicting situations and/or may face conflicts without a principle of arbitration and solution in the short and long term. Conversely, findings from developmental psychology demonstrate that in humans abstract cognitive skills—such as the use of abstract verbs or numerical reasoning—are fundamentally grounded in sensorimotor activity, and bodily experience scaffolds symbol formation. Such a process allows a smooth integration of first-person and third-person knowledge, as suggested by computationally modeled formulations (Cangelosi and Stramandinoli, 2018).

The foundational role of the body becomes particularly evident when we contrast it with the limitations of current large-scale learning architectures. Despite their remarkable performance in language and vision tasks, foundational models—whether LLMs or VLMs—struggle to generalize seemingly trivial spatial concepts. Notions such as height, relative position, or reachability—intuitively mastered by humans from early infancy through bodily interaction—remain elusive for these models, especially when they are applied in robotic contexts. For instance, robotic arms trained through behavioral cloning on extensive demonstrations show impressive proficiency when reproducing known actions in familiar settings. However, even minor changes—such as a slight shift in the object’s position, a different tablecloth pattern, or a slight variation in height—can cause performance to drop dramatically, as recently pointed out by Dieter Fox in his “Where’s RobotGPT” talk (Fox, 2024). These changes, which humans would effortlessly generalize due to their embodied spatial understanding and reasoning, often require retraining or additional examples for the model to adapt, thus illustrating how the lack of embodied grounding and causality severely limits the generalization and adaptability of current AI models in physical environments.

A fundamental difference between passive learning—as performed by LLMs—and the kind of first-person learning we advocate lies in the nature of captured relationships. LLMs, trained on vast corpora of linguistic data, have proven remarkably capable of extracting statistical regularities, many of which reflect deep correlations embedded in language. This correlational power allows them to solve complex tasks with apparent fluency. However, this mechanism radically differs from how humans (and robots with a fundamentally embodied training experience) learn through active engagement with the world. In first-person learning, the agent does not merely observe correlations—it experiences *causation*. By performing an action with a specific goal in mind and perceiving its consequences, the agent can establish a direct link between its behavior and the resulting outcome. This ability to isolate causal mechanisms provides a powerful filter that distinguishes meaningful action-effect relationships from coincidental correlations, a fundamental process well documented in the developmental literature. According to the “interventionist” view of causality (Gopnik and Schulz, 2007), knowing that X causes Y implies that manipulating X leads to a change in Y. Children learn about causation precisely through intentional interventions and the observation of contingent outcomes. By age four, children can actively experiment to infer causal structures (Schulz and Bonawitz, 2007), going beyond early Piagetian learning, where actions are simply associated with their direct outcomes (Piaget, 1930). Even at around 24 months, infants are adept at observational causal learning: they do not merely imitate or detect correlations between events but infer causal relationships from others’ actions and use those inferences to plan their own interventions (Meltzoff et al., 2012). These findings suggest that the human capacity for causal learning is rooted in embodied, intentional activity from a very early age. Crucially, this causally grounded understanding—emerging from both action and observation—enables robust generalization. Instead of brute-force pattern matching over all conceivable correlations, the agent can reason about what actions are likely to produce desired effects, even in unfamiliar scenarios. Replicating this capacity in artificial agents requires grounding learning in embodied, interactive experience. Otherwise, the ability to generalize causal knowledge across domains and contexts will likely remain severely limited.

Moreover, it may be observed that the computational model, which forms the philosophical foundation of artificial intelligence, implies intractable problems (Clark, 1999): in particular, an information bottleneck occurs when the mind is requested to construct detailed representations of the external world to produce appropriate purposive actions. The problem is that the world is constantly changing, for its dynamics and as an effect induced by the agent’s actions, and thus, the demands on the mental system are likely to preclude the agent from producing appropriate actions just in time. The nature of such information bottleneck, due to the supposed need for a multiverse representation of the environment, is another aspect of the computational prodigality that characterizes AI in general and EAI in particular, based on the “brute-force” assumption that infinite amounts of training data are available and computational resources are vast and free. In contrast, as observed by Clark, humans need relatively little information about the world before they manage to act effectively upon it.

Vision and, in general, the multi-sensory perception of the peripersonal space in the surrounding environment (Di Pellegrino and Làdavas, 2015; de Vignemont et al., 2021) is an active, purposive, attention-driven process not a passive, high-resolution, virtual representation. Although the spatial awareness implicit in everyday life supports the illusion of a stable and fully detailed representation of the world, this subjective impression (Clark, 1997) obscures the reality of minimal and low-detail environmental information where the constraint of quick action guides the search and acquisition of missing perceptual evidence to extract information “just in time.” This concept exemplifies the computational frugality of the bidirectional human embodied cognition. It avoids the computationally expensive reconstruction of a detailed world model on the working assumption that the world is its best model, which only needs to be sampled where and when required.

Another crucial aspect of the computational frugality of the human-embodied cognitive system is related to the role of episodic memory (Dickerson and Eichenbaum, 2010) in the framework of versatile and articulated human memory systems. The episodic memory system is implemented in the brain by an extended circuitry centered around the medial temporal lobe (MTL), interacting with several cortical and subcortical areas: the functions of the cortical components address many aspects of perception and cognition, whereas the MTL system mediates the formation and retrieval of the associative network of memories whose details are stored in the cortical areas. Episodic memories (EMs) are related to specific personal experiences that occur in daily life and, for some reason, are isolated for their “exceptional” relevance and stored in long-term memory. The motivations to single out such episodes from the sensory-motor flow of daily life can be of different types, such as curiosity, novelty detection, emotional drive, social interaction with a teaching master, etc. These memories are structured chunks of information that include spatio-temporal patterns about sequences of actions and the surrounding environment. They include a declarative component, expressed explicitly by direct conscious access to information and communicated by spoken language, and a nondeclarative component, such as a procedural memory about the learned sequence of movements or actions appropriate for the memorized episode.

Episodic memories are unique samples extracted from any embodied cognitive agent’s continuous sensorimotor experience flow and coded in some associative storage. Sensorimotor intelligence implies the dual capacity, on one side, to identify and code relevant or crucial episodes and, on the other, to detect the resonance with a stored episode in a given action sequence. Thereafter, the cognitive agent is supposed to quickly retrieve the detailed episode, adapt it to the specific circumstance, and produce the corresponding procedural behavior. In any case, this mnemonic process is not reproductive, as a kind of playback routine of stored information, but reconstructive, namely the activation of an internal simulation model based on the key parameters stored in the episodic memory. Of course, episodic memories are far from detailed digital recordings and do not need to be so. When retrieved from long-term memory to guide an action plan, they are instantiated with slight modifications induced by the situation and the agent’s state. However, this kind of flexibility may include margins of failure if the recall occurs in extreme conditions. This issue is well known in forensic psychology (Sarwar et al., 2004) concerning the possible contradictions of eyewitnesses: due to the reconstructive nature of the mnemonic process, it is likely that the event recalled by an eyewitness, in situations with intense emotional stress, is corrupted by unrelated memory fragments that have nothing to do with the truth. Such a problem affects large associative memory systems, such as Hopfield networks (Hopfield, 1982), in case of overloading. However, this does not affect the main issue, namely the fact that the episodic memory system, in association with procedural memories, is a formidable mechanism of computational frugality that allows a cognitive agent to store and retrieve the minimum amount of information together with the minimum amount of computational power.

Preliminary studies have explored the systematic use of episodic memories in cognitive robotics (Mohan et al., 2014; Vernon et al., 2015). In our opinion, this is one of the crucial research lines to be further investigated in embodied cognitive robotics. The main difference of this approach, in comparison with the EAI approach, based on large foundation models, is that it is based on first-person experience rather than third-person, pre-coded knowledge: computational frugality of the single autonomous agent vs. computational prodigality of the population of super-intelligent agents. However, this does not imply that cognitive robots, educated according to principles of first-person acquisition of experience, cannot take advantage of the consultation of encyclopedic knowledge stored in books, manuals, movies or web browsers using language tools as AI foundation models. This (third-person) knowledge can be used to update or adapt/consolidate first-person know-how obtained through personal experience, for example, by modifying specific parameters of episodic memory or the associated procedural trace. The opposite process, namely integrating first-personal knowledge into a large third-person structure, is unnatural and impractical.

Along the same line of reasoning, we suggest that the issue of computational frugality can be associated with the well-known epistemological, philosophic concept known as Ockham’s razor, namely the principle of cognitive parsimony in the search for an explanation of scientific or philosophic problems, i.e., the principle that robust explanations should be constructed with the smallest possible set of elements: entia non sunt multiplicanda praeter necessitate (entities should not be multiplied beyond necessity).

## Embodied cognition and the extended mind hypothesis

In a previous section, we briefly discussed the extended mind hypothesis (Clark and Chalmers, 1998), concerning the organization of embodied cognition, and we suggested two interrelated extensions: integration with the physical environment and integration with the social environment. The former issue deals with the need for the embodied cognitive system to incorporate some degree of what is known as commonsense knowledge about the dynamics of the physical world, including causality during physical interaction, the effect of gravity, etc. Commonsense representation and reasoning have been among the central issues addressed by symbolic AI (a.k.a. GOFAI: Good Old-Fashioned AI) in the 70s and 80s, focused on a family of computational models known as expert systems. In particular, the subsets of expert systems oriented to robotic applications were designed to implement *qualitative physics* (Forbus, 1988): the key idea was to find ways to represent continuous properties of the world, traditionally formulated employing differential equations, by discrete systems of symbols, thus allowing different styles of reasoning, like qualitative simulation and envisioning. The success of this approach to commonsense was somewhat limited, with scarce application to autonomous robotics. More recently, with the expansion of connectionist AI to the level of foundation models, the topic was revisited under the label of intuitive physics (Piloto et al., 2022): it is conceived as a network of concepts focused on the discovery of the hidden principles that explain the interactions of macroscopic objects in the real world. The suggested approach is to use foundation models of the VLMs type, such as the PLATO (Physics Learning through Auto-encoding and Tracking Objects). PLATO is a foundation model that has been trained by a large number of videos depicting objects interacting according to the laws of physics. For simplicity, the videos were generated by simulation experiments, not by observed phenomena. The big dataset was used to train large, deep networks to acquire some commonsense understanding of the dynamics of the physical world, which is useful for reasoning and prospection.

In a different way and with a different sophistication level, qualitative physics, and intuitive physics models fail to achieve the goal of EAI, namely the design of autonomous, intelligent, collaborative robots because are unable to implement an embodied cognitive architecture fully integrated with the first-person experience of a cognitive agent operating in a specific environment and with well-defined functions. The alternative to PLATO is bio-inspired in the sense of exploiting the simulation theory of cognition, which supervises both real and virtual sensorimotor patterns performed in the context of the current world model and of the typical tasks performed by the cognitive agent in real life and cooperation with human or robotic partners. Thus, the training data that are necessary for developing or updating neural models capable of achieving an intuitive understanding of physics are self-generated by the cognitive agent: intuitive physics and/or other intuitive understanding of the dynamics of the environment and the body-environment interaction and are implicitly addressed in a first-person manner. Moreover, this first-person approach combines active synergy formation with data preparation for learning, including the critical labeling step, an Achilles’ heel for training foundation models.

The intuitive physics component of the extended mind hypothesis, based on the active physical interaction with the environment, may also be characterized as a computationally frugal strategy, accumulating an amount of data for self-training according to the principle as much as needed, not in general but in a personalized way for the specific cognitive agent.

In a bio-inspired way, we suggest that the extension of the extended mind hypothesis as a result of learning by self-training is the result of a developmental process, organized in layers according to the Piagetian theory with a progressive acquisition of the level of intuitive physics understanding. We may also envisage that, at high levels of cognitive capability, including a sufficient level of linguistic competence, the cognitive agent may be motivated to search for third-person knowledge by interrogating commercially available foundation models trained with massive encyclopedic datasets. For example, the answer provided by the foundation model may allow the cognitive agent to choose one alternative sequence of actions among several possibilities consistent with his previous experiences and incorporate this information in the corresponding episodic memory.

As regards the social extension of the extended mind hypothesis, we should consider the broad research area at the border of motor neuroscience and cognitive neuroscience, namely the large number of experimental studies that emphasize the strong implication of the motor system, specifically responsible for the production of covert and overt actions, also in typical cognitive functions as action observation, imitation and social interaction (Fadiga et al., 1995; Fadiga et al., 2002; Iacoboni et al., 1999; Grezes et al., 2001) as well as activities related to movement in a more abstract way as the observation of manual tools or the use of action verbs (Martin et al., 1996; Grafton et al., 1997). Although these studies were aimed primarily at understanding the interaction of human subjects with the environment and/or the interaction between humans, we believe that they can be naturally extended to the design of autonomous, intelligent, and collaborative robots.

We can view the motor and cognitive systems as forming a pair of equivalent loops, one related to open actions and the other to covert actions: in the former case, motor commands cause muscle contractions with consequent sensory feedback, which in turn influences the control of future motor commands; in the latter case, motor intentions activate an internal body schema with consequent sensory predictions, which in turn affect the ideomotor formulation of future action plans (Mohan et al., 2019). When a person (a naïve performer) interacts with another person (an expert), we can think of an analogous additional loop, namely a social interaction loop in which the “controlled object” is the other person rather than the actual or imagined motion of one’s own body. For example, the social interaction loop is instantiated because the naïve person attempts to imitate the expert or because the experts supervise the naïve partner’s action, guiding his performance with intermittent intervention. Therefore, in social interactions, by controlling someone else rather than our own body, we can estimate their hidden state, including their mental state, rather than our own body (Wolpert et al., 2003). In other words, the control signals that characterize social interactions may be considered communicative actions, including speech, gestures, and haptic interaction.

One of the primary motivations for adopting this approach for a fully embodied AI roadmap is that it strongly matches the requirement of mutual understanding between humans and robot partners in a very general sense. Equipping robots with a cognitive architecture grounded in first-person experience allows the emergence of a form of cognitive compatibility between human and robot agents. When both share similar developmental principles—such as the incremental accumulation of sensorimotor experiences, episodic memory formation, and action-oriented reasoning—the human partner is more likely to understand the rationale behind the robot’s behavior, including its mistakes. This compatibility can play a crucial role in enabling a more natural and effective form of robot education (Matarese et al., 2021). In such a scenario, humans can more easily interpret the robot’s errors and provide corrective feedback in ways that the robot can meaningfully incorporate. As a result, robot learning becomes more transparent, interpretable, and ultimately more efficient. Conversely, when it is the robot’s turn to provide suggestions or assistance, its behavior is more likely to be perceived as understandable and trustworthy (Matarese et al., 2023). This compatibility not only facilitates the correction of sensorimotor mistakes or the refinement of practical know-how but also opens the door to a richer educational process involving abstract and culturally embedded concepts, including notions of what is considered appropriate or inappropriate behavior. In other words, it becomes feasible to teach a robot not only how to do something, but also whether it should be done—based on the teacher’s values. Much like a child learns through example and imitation what is right or wrong within a given family or culture, a robot equipped with an embodied cognitive architecture may become receptive to similar forms of moral or normative guidance (Sandini et al., 2024). Even in the case of interspecies learning, such as training a dog, the process succeeds despite evident communicative asymmetries because the animal learns through embodied interaction and contextual reinforcement. Similar dynamics can be envisioned in human-robot interaction, provided that the robot cognition is grounded in first-person experience and can structure knowledge accordingly. In contrast, this possibility is largely inaccessible in the case of passive AI systems trained offline on abstract, third-person data, where the encoding of universal ethical rules becomes an ill-defined and arguably unsolvable problem.

Moreover, this is also the roadmap for integrating learning through first-person experience and third-person interaction with various interaction channels, from web-interrogation to human education and tutoring. The relevance of the educational issue of embodied cognition (Hegna and Ørbæk, 2021) is also thoroughly addressed by a theme issue “Minds in movement: embodied cognition in the age of artificial intelligence” of the Philosophical Transactions B (Barrett and Stout, 2024). After having recognized embodiment as a unifying concept in the study of cognition, this study focuses on two key themes, namely the role of language in cognition and its entanglement with the body and the multiple bodily mechanisms of interpersonal perception and alignment across the domains of social affiliation, teaching, and learning: in both themes, AI language models can be valuable tools for robot training.

## Conclusion

Summarizing this opinion paper, we may say that the roadmap to the design of autonomous, intelligent, collaborative robots supposed to infiltrate our society for its expected technology-driven reorganization can be characterized according to the following principles:

- Fully embodied cognitive architecture functionally equivalent to the human counterpart
- Learning and training based on prospection capabilities and accumulation of first-person experiences stored in episodic memory
- The crucial role of social interaction for accessing third-person information, defining the intelligence level appropriate for the sought performance target and achieved through a process of sensory-motor-cognitive development
- Human-robot collaboration should adhere to the principle that the limit to a robot’s autonomy is the ultimate responsibility of the human partner and/or the social environment at large.

In general, we suggest that it does not make sense (from the scientific and economic sense) to aim at a single design target of a super-intelligent robot to be easily adapted to the variety of application paradigms that should fit social needs. In many situations, this could be too much and a waste of computational resources; in specific situations, it could be too little. We suggest a frugal computational architecture with an initial, minimal configuration to be grown up through learning and training in a well-organized social context. Such minimal architecture could be conceived as an extension of the Vehicle paradigm (Braitenberg, 1984), grown up in a self-organizing and self-training context. Moreover, we fully agree with (Lake et al., 2017) that such machines should be designed to learn and think like people.

An alternative roadmap is pursued by AI companies, counting on the continuously growing progress in developing LLMs to understand human requests and communicate plans of action using natural language. A very recent example is provided by the new model, Gemini Robotics, developed by Google DeepMind (Gemini Robotics Team, Google DeepMind, 2025) that combines its best large language model with robotics. The goal is to give robots the ability to be more dexterous and generalize across tasks, exploiting the generalizing capabilities of LLMs, such as reasoning about which actions to take in a given context. In any case, Gemini robots are trained similarly to most LLMs, namely text, images, and videos from the internet or synthetic data generated by simulation models without any personal accumulation of personal experience and first-person knowledge. The explicitly stated rationale of the Gemini Robotics family is to develop general-purpose robots that realize AI’s potential in the physical world: despite the remarkable documented examples of performance, we believe that this is not the appropriate roadmap for the massive diffusion of cooperative cognitive agents in a variety of qualitatively different scenarios as robot teachers, robot helpers, robot companions, and so on. Critical features of such scenarios may not be coded in large collections of text, images, videos, or synthetic datasets but may be hidden in haptic interaction, haptic guidance, and gestural non-verbal communication with human partners, thus allowing the crucial development of shared embodied cognition of robot and human cooperative partners.

The research group that includes the authors of this paper has been working on various building blocks for the design of autonomous, intelligent, collaborative robots according to a bio-inspired roadmap based on embodied cognition for more than a decade. In particular, this research activity has been focused, among other things, on prospection, learning a body schema, embodied simulation of action, imitation learning, episodic memory, understanding physical interaction, social cognition based on embodied communication, and developmental learning (Lungarella et al., 2003; Mohan and Morasso, 2007; Metta et al., 2010; Mohan et al., 2011, 2013, 2014; Vernon et al., 2015; Bhat et al., 2016; Bhat et al., 2017; Sciutti and Sandini, 2017; Sandini et al., 2018, 2024; Pasquali et al., 2025). We are still away from an implementation framework that allows us to integrate the variety of building blocks outlined above in a flexible and self-organizing way. In our opinion, such a framework should be hybrid, combining digital, analog, symbolic, and subsymbolic representations similar to what we know of the human embodied cognitive architecture. In any case, we are confident that the proposed roadmap is naturally suitable for facing ethical issues and social impact because the main design goal is to facilitate a shared embodied cognition between the robot and the human companion as much as possible.
